# Prevalence of hypopituitarism after intracranial operations not directly associated with the pituitary gland

**DOI:** 10.1186/1472-6823-13-51

**Published:** 2013-11-04

**Authors:** Steffen Kristian Fleck, Henri Wallaschofski, Christian Rosenstengel, Marc Matthes, Thomas Kohlmann, Matthias Nauck, Henry Werner Siegfried Schroeder, Christin Spielhagen

**Affiliations:** 1 Department of Neurosurgery, University Medicine Greifswald, Ferdinand-Sauerbruch-Strasse, 17475 Greifswald, Germany; 2 Institute of Clinical Chemistry and Laboratory Medicine, University Medicine Greifswald, Ferdinand-Sauerbruch-Strasse, 17475 Greifswald, Germany; 3 Institute of Community Medicine, University Medicine Greifswald, Ferdinand-Sauerbruch-Strasse, 17475 Greifswald, Germany

**Keywords:** Hypopituitarism, Pituitary deficiency, Intracranial operation, Neurosurgery

## Abstract

**Background:**

Over the last few years, awareness and detection rates of hypopituitarism following traumatic brain injury (TBI) and subarachnoid hemorrhage (SAH) has steadily increased. Moreover, recent studies have found that a clinically relevant number of patients develop pituitary insufficiency after intracranial operations and radiation treatment for non-pituitary tumors. But, in a substantial portion of more than 40%, the hypopituitarism already exists before surgery. We sought to determine the frequency, pattern, and severity of endocrine disturbances using basal and advanced dynamic pituitary testing following non-pituitary intracranial procedures.

**Methods:**

51 patients (29 women, 22 men) with a mean age of 55 years (range of 20 to 75 years) underwent prospective evaluation of basal parameters and pituitary function testing (combined growth hormone releasing hormone (GHRH)/arginine test, insulin tolerance test (ITT), low dose adrenocorticotropic hormone (ACTH) test), performed 5 to 168 months (median 47.2 months) after intracranial operation (4 patients had additional radiation and 2 patients received additional radiation combined with chemotherapy).

**Results:**

We discovered an overall rate of hypopituitarism with distinct magnitude in 64.7% (solitary in 45.1%, multiple in 19.6%, complete in 0%). Adrenocorticotropic hormone insufficiency was found in 51.0% (partial in 41.2%, complete in 9.8%) and growth hormone deficiency (GHD) occurred in 31.4% (partial in 25.5%, severe in 5.9%). Thyrotropic hormone deficiency was not identified. The frequency of hypogonadism was 9.1% in men. Pituitary deficits were associated with operations both in close proximity to the sella turcica and more distant regions (p = 0.91). Age (p = 0.76) and gender (p = 0.24) did not significantly differ across patients with versus those without hormonal deficiencies. Groups did not significantly differ across pathology and operation type (p = 0.07).

**Conclusion:**

Hypopituitarism occurs more frequently than expected in patients who have undergone neurosurgical intracranial procedures for conditions other then pituitary tumors or may already exists in a neurosurgical population before surgery. Pituitary function testing and adequate substitution may be warranted for neurosurgical patients with intracranial pathologies at least if unexplained symptoms like fatigue, weakness, altered mental activity, and decreased exercise tolerance are present.

## Background

Evaluation of endocrine function plays an essential role in pituitary tumor diagnostics. Even in experienced neurosurgical centers, high rates (ranging from 80-100%) of postoperative pituitary deficiencies have been reported. Furthermore, patients who have received radiation or chemotherapy or are suffering from childhood tumors, craniopharyngeomas, or skull base tumors carry a high risk of developing hypothalamo-hypophyseal disturbances [1,2].

Studies assessing posttraumatic hypopituitarism have only recently been published. However, earlier postmortem studies identified anterior gland necrosis in up to one third of fatal head injuries and several case reports of posttraumatic hypopituitarism exist [3,4].

Today, endocrinologists and neurosurgeons are becoming increasingly aware of the relationship between traumatic brain injury (TBI) and pituitary dysfunction [3,5-10]. However, subarachnoid hemorrhage (SAH) has also been linked with neuroendocrine dysfunction in a substantial number of patients, possibly indicating the need for endocrinology follow-up evaluations in these patients as well [11,12].

In published series, the rate of patients with hormone deficits of the hypothalamo-pituitary axis varies, ranging from 20 to 80%. These disturbances may have an important influence on health status, neurobehavioral complaints, and rehabilitation potential. Because hormonal deficiencies can develop either acutely or long after (months to years) TBI or SAH, a neuroendocrine follow-up period might be necessary to determine if hormonal replacement is necessary [5].

Schneider et al. assessed endocrine abnormalities in 68 patients who underwent surgery for non-pituitary intracranial tumors while also receiving chemotherapy or radiation and found that over 40% of all patients had hormone irregularities [13]. However, studies investigating the frequency of pituitary insufficiency after intracranial operations for non-pituitary tumors are limited [14,15] which highlight the need of more data. Therefore, we sought to prospectively determine the frequency of hypopituitarism and hypopituitarism-related factors in postoperative patients using basal parameters and advanced pituitary function tests.

## Methods

### Patients

This study was conducted prospectively at the Greifswald University of Medicine and was approved by the investigational and ethical review board of the University of Greifswald. Informed consent was obtained from all patients.

51 consecutive patients were eligible for enrollment having undergone a neurosurgical intracranial procedure for something other than a pituitary tumor (29 female, 22 male, mean age 55 years, age range 20 to 75 years), (BMI mean 27.9; range of 21 to 37).

Basal hormone status and pituitary function testing was performed between 5 and 168 months (mean 47.2) after the operation. Two patients were excluded from the adrenocorticotropic function testing due to an ongoing glucocorticoid replacement therapy.

### Localization of tumor/approach

The tumor or approach localization was divided into subgroups in accordance with Schneider et al. [13] (with the exception of medial and lateral sphenoid wing processes). With regard to proximity to the hypothalamic-hypophyseal region, the subgroups were characterized as, (1) central: medial sphenoid, clinoid, tuberculum sellae, intra-/supra-/parasellar, third ventricle; (2) frontal: lateral sphenoid, frontal, fronto-temporal, fronto-parietal, frontobasal, ethmoid; (3) temporal/parietal: parieto-occipital, lateral ventricles, petrous, petroclival, acoustic nerve; and (4) occipital: occipital, cerebellar, pineal, fourth ventricle.

We additionally recorded the occurrence of hydrocephalus accompanying the operation (endoscopic third ventriculostomy (ETV), ventriculo-abdominal (VP) shunt), epilepsy, complications in the postoperative course, and radiation/chemotherapy.

### Evaluation of pituitary function

All patients were tested following an overnight fast. The laboratory analytics were measured at the Institute of Clinical Chemistry and Laboratory Medicine at the University Medicine, Greifswald. The following basal measurements were performed. At 8:00 AM, we measured serum cortisol (adrenal insufficiency as indicated by < 100 nmol/l), free thyroxin (fT4), thyroid-stimulating hormone (TSH), insulin-like growth factor-I (IGF-I), follicle-stimulating hormone (FSH), luteinizing hormone (LH), and testosterone (men). Moreover, we evaluated urine and plasma sodium, osmolality, and diuresis for 24 hours. Patients underwent the following dynamic testing.

Insulin tolerance test (ITT): we performed an ITT to assess cortico- and somatotrophic secretion. In this test we measured adrenocorticotropic hormone (ACTH), cortisol, and growth hormone (GH). 0.1-0.15 IE insulin/kg body weight (BW) were intravenously administered at 0 min to induce a fall in blood glucose level to < 3 mmol/l and neuroglycopenia symptoms. ACTH, cortisol, GH, and blood glucose levels were measured at -15, 0, +30, +60, +90, and +120 minutes. In cases, where an ITT was contraindicated, a combined growth hormone releasing hormone (GHRH)/arginine test and a low dose ACTH test were performed.

Combined (GHRH)/arginine test: GHRH was given (1 μg/kg i.v. at 0 min) along with L-arginine hydrochloride 6% (i.v. infusion over 30 min from 0 to + 30 min at dosages of 0,5 g/kg, maximum 30 g). GH levels were measured at -15, 0, +30, +60, +90, and +120 minutes.

Low dose ACTH test: 1 μg ACTH (synacthen®) i.v. was injected at 0 min and cortisol was measured -15, 0 min and after 30 and 60 min.

Twenty-one patients underwent ITT, thirty patients underwent low dose ACTH test, and all patients underwent GHRH/arginine test.

### Definitions of pituitary deficiencies

Pituitary deficiencies were diagnosed with respect to the clinical symptoms and result of the dynamic testing. Before study start criteria have been defined as follow:

Growth hormone deficiency (GHD): GH peak after ITT < 5 μg/l or GH peak after GHRH + Arginine test < 16 μg/l [16]. In cases where a GHRH/arginine test was performed, body mass index (BMI) dependent cut-offs were used [17]. Partial GHD was defined as patients with IGF-I levels within the age- and sex-related reference range and a pathological ITT or GHRH/arginine test. We defined severe GHD as a GH peak after ITT < 3 μg/l [18] or a GH peak following the GHRH/arginine test < 11 μg/l if BMI was < 25 [17].

Corticotrophic deficiency: was defined as baseline cortisol < 100 nmol/l [19] or cortisol < 550 nmol/l following the ITT or low dose ACTH test. Partial adrenocorticotrophic insufficiency was diagnosed if a twofold increase in baseline cortisol (above 100 nmo/l) was observed but overall cortisol levels remained lower than 550 nmol/l.

Thyrotrophic deficiency: was detected if fT4 were below the lower reference range [11,12,20] in combination with an inappropriately normal or low normal TSH. That means TSH is within the reference range and not increased as expected for primary hypothyroidism.

Hypogonadism: in men was defined as low testosterone serum concentration (< 10 nmol/l) in combination with inadequate low gonadotropins (LH, FSH) [21]. We defined hypogonadism in premenopausal women as oligo- and amenorrhea, and as low gonadotropins in postmenopausal women.

Diabetes insipidus centralis: was identified by the presence of an increased volume of dilute urine (> 6 l/24 hours) with low urine osmolality (< 300 mosmol/kg).

### Statistical analysis

We performed Fisher’s exact tests to assess comparisons between groups. Two-tailed p-values of <0.05 were considered significant. We conducted all analyses with SPSS 16.0 (SPSS Inc., Chicago, IL). Furthermore, we calculated 95% confidence intervals according to Altman et al. [22].

## Results

We included and evaluated endocrine function in 51 patients (29 female, 22 male; mean age 54.9 years, range 20–75 years; BMI 27.9, range 21–37). Localization of intracranial pathologies included the (1) central (n = 10), (2) frontal (n = 9), (3) temporo-parietal (n = 20), and (4) the occipital regions (n = 12).

22 patients had a meningeoma, 4 patients had an astrocytoma, 3 a vestibular schwannoma, 3 a cavernoma, 3 a brain abscess, 3 had dural arterio-venous fistula, 2 had ependymomas, 1 patient had a pinealoma, 1 an oligodendroglioma, 1 a ganglioglioma, 1 a haemangioblastoma, 1 had trigeminal nerve decompression, 1 cavum vergae, 1 patient had epilepsy surgery, 1 had an aneurysm (without SAH), 1 had an arterio-venous malformation, and finally 1 patient had a subdural hematoma. To compare pathology subgroups we categorized diagnoses as follows:

(a) meningeomas (n = 22);

(b) astrocytoma, glioblastoma, oligodendroglioma, ganglioglioma (n = 7);

(c) vestibular schwannoma, ependymoma, pinealoma (n = 7);

(d) vessel associated pathologies (9);

(e) others: subdural hematoma, cavum vergae, epileptic lesion (n = 3)

(f) brain abscess (n = 3).

Accompanying hydrocephalus occurred in five patients and a history of epileptic seizures in eight patients. Six patients underwent postoperative radiation (combined with chemotherapy in two patients). One patient developed postoperative meningitis, which resolved completely following antibiotic therapy.

**Figure 1:**
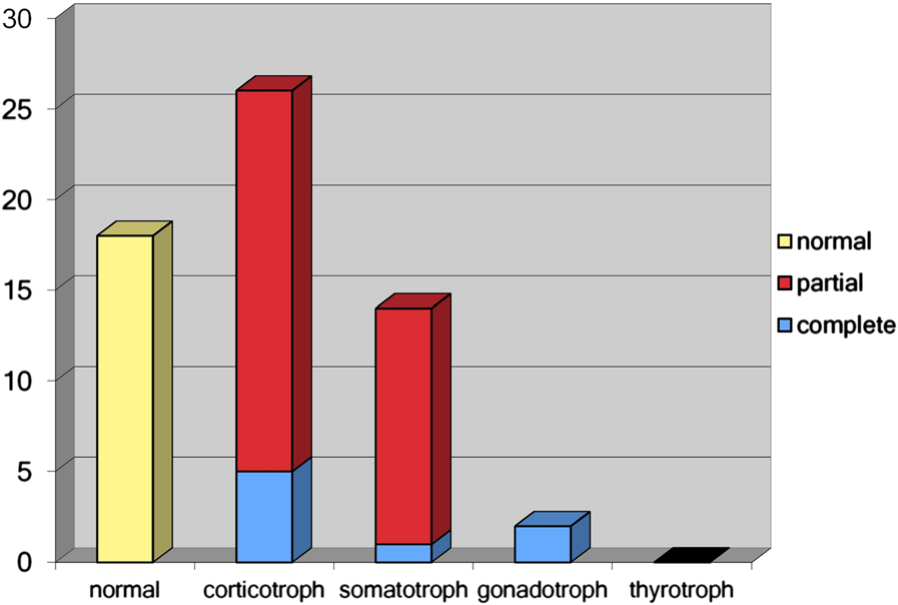
Frequency of pituitary deficiencies (gonadotrophic function in men).

As illustrated in Figure 1, a total of 35.3% of patients showed no hormonal abnormality.

**Figure 2:**
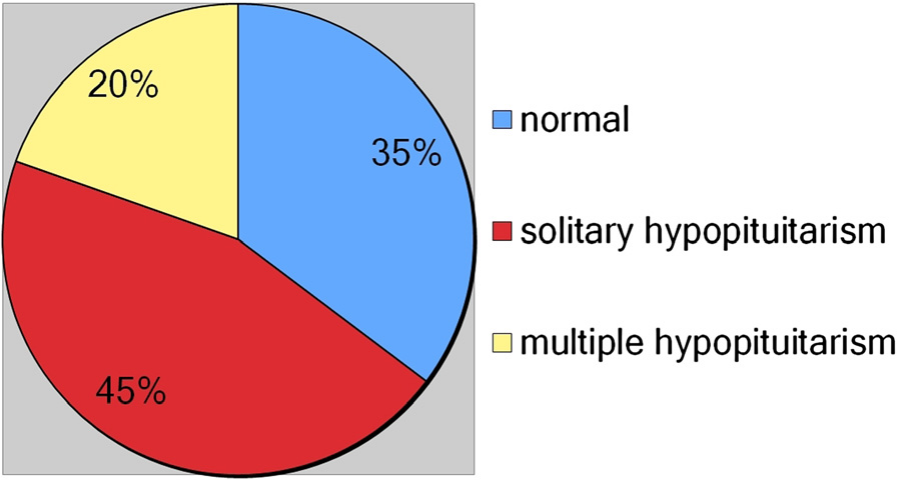
Frequency of pituitary deficiencies.

Overall, we detected solitary hypopituitarism in 45.1%, multiple hypopituitarism in 19.6%, and complete insufficiency in 0% (see Figure 2).

Adrenocorticotrophic deficiency, in varying degrees, was observed in 51.0% of patients (partial 41.2%, complete 9.8%). No patient in the present study had baseline cortisol < 100 nmol/l, therefore, we could not identify any patient to have secondary adrenal insufficiency as indicated by the basal cortisol level only. The diagnosis of adrenocorticotrophic deficiency in all patients is provided by dynamic testing. 31.4% of patients had a GHD (partial 25.5%, severe 5.9%), whereas a thyrotrophic deficiency was not observed. The prevalence of gonadotrophic deficiency was 9.1% (n = 2) in men (see Figure 1). In case of the usage of oral contraceptives or hormone replacement therapy in majority of women hypogonadism could not be exactly differentiated. Two patients developed diabetes insipidus in the early postoperative stage. Diabetes insipidus was not detected at the time of endocrine follow-up.

We observed no tendency toward a lower frequency of observed adrenal insufficiency in patients tested at longer intervals after surgery (time points 24 (p = 0.4654) and 48 months (p = 0.1441).

According to pathology localization, we classified endocrine abnormalities (complete and partial) as illustrated in Tables 1 and 2. Fischer’s exact tests revealed that there was no association between endocrine abnormalities and surgical approach/pathology location (p = 0.91).

**Table 1:** Hormonal deficiencies according to localization of tumor/approach

***Region***	***Patients***	***Deficiencies***	***Confidence interval (95%)***
	***(n)***	***% (n)***	
**(A) “Central”**	10	70.0% (7)	0.7 (0,40-0.89)
**(B) “Frontal”**	9	55.5% (5)	0.56 (0.27-0.81)
**(C) “Temp.par.”**	20	60.0% (12)	0,60 (0.39-0.78)
**(D) “Occipital”**	12	66.6% (8)	0.67 (0.39-0.86)
***Overall rate***	***51***	***62.7% (32)***	

**Table 2:** Deficiencies of specific axis according to localization of tumor/approach

***Region***	***Patients***	***Adrenoc. deficiency***	***Growth hormone deficiency***	***Thyreotroph deficiency***	***Gonadotroph deficiency***
	***(n)***	***% (n)***	***% (n)***	***% (n)***	***% (n)***
**A: “Central”**	10	70% (7)	50% (5)	0	10% (1)
**B: “Frontal”**	9	44.4% (4)	22.2% (2)	0	0
**C: “Temp.par.”**	20	50% (10)	15% (3)	0	0
**D: “Occipital”**	12	41.7% (5)	33.3% (4)	0	8.3% (1)
***Overall rate***	***51***	***50.9% (26)***	***27.4% (14)***	***0***	***9.1% (2of 2men)***

An adrenocorticotrophic deficiency (partial or complete) was observed in two of the five patients with accompanying hydrocephalus. Of these patients, a GHD was detected in one patient, but none were found to have a thyrotrophic or gonadotrophic deficiency.

Of the eight patients with a history of seizures, seven patients (87.5%) exhibited hypopituitarism along the adrenocorticotrophic axis and 25% (two patients) along the somatotrophic axis. Thyrotrophic and gonadotrophic deficiencies were not detected in patients with epilepsy. The difference between those patients with and without epileptic seizures was significant (p = 0.018).

Six patients received radiation and/or chemotherapy. The rate of hypopituitarism (any form) did not significantly differ across patients with versus those without radiation and/or chemotherapy (p = 0.639).

No associations were found between endocrine deficiencies (binary grouping: yes/no), age (binary grouping: ≤50, >50 years; p = 0.757), gender (p = 0.236), and tumor presence (binary variable: yes/no; p = 0.761). Furthermore, pathology/operations were not associated with endocrine abnormalities (p = 0.0715; see Table 3).

**Table 3:** Hormonal deficiencies according to entities

***Entities***	***Pat.***	***Deficiencies***	***Confidence interval (95%)***
	***(n)***	***% (n)***	
**(A) Meningeomas**	22	54% (12)	0.54 (0.35-0.73)
**(B) Astrocytoma, glioma, oligodendroglioma, ganglioglioma**	7	86% (6)	0.86 (0.49-0.97)
**(C) Vestibular schwannoma, ependymoma, pinealoma**	7	57% (4)	0.57 (0.25-0.84)
**(D) Vessel associated diseases**	9	88% (8)	0.88 (0.56-0.98)
**(E) Subdural hematoma, epileptic lesion, Cavum vergae**	3	66% (2)	0.66 (0.21-0.94)
**(F) Brain abscess**	3	0.00% (0)	0.00 (0.00-0.56)
***Overall rate***	***51***	***62.7% (32)***	

## Discussion

The awareness and detection rate of hypothalamo-pituitary hormone disturbances has greatly improved over the last decade, illustrating their high frequency after TBI, SAH, radiation, and neurosurgical procedures (for reasons other than pituitary tumors) [23]. Consequently, interdisciplinary expert recommendations regarding endocrine evaluation in SAH and TBI patients have been published [8,24]. The primary finding of this study is that hypopituitarism occurs more frequently than previously expected in patients who have undergone non-pituitary related intracranial procedures.

Our results are in accordance with Schneider et al. and De Marinis et al., who also found that a clinically relevant number of patients develop pituitary insufficiencies following non-pituitary operations [13,14]. However, our patient sample differs from that of Schneider et al., who evaluated 68 consecutive patients who underwent operations for non-pituitary intracranial tumors due to clinical suspicion of hormone deficiencies [13]. We detected mainly single axis disturbances (45.1%), followed by multiple axis disturbances (19.6%), and did not find any patients with total insufficiency (see Figure 2). Studies assessing endocrine disturbances in TBI and SAH patients have also primarily reported finding single axis deficiencies [12,25,26].

It has been suggested that screening for hypopituitarism (performed within 21 days after brain injury as well as 12 weeks and 12-month postoperative) is necessary [26]. We performed testing between 5 and 168 months (mean 47.2) after the procedure to evaluate possible longstanding hormonal deficiencies closer to the clinical practice. Wachter et al. [15] postulated that pituitary insufficiencies are already present before surgery but they performed the evaluation only 1 and 7 days after surgery, which might have the potential to overestimate the frequency of hypopituitarism in critical illness.

Our patients received hormone replacement therapy if hypopituitarism was diagnosed. Patients with severe GHD are eligible for GH replacement therapy to normalize disturbances associated with adult GHD [8]. Generally, however, neurosurgical patients are not routinely considered for endocrine evaluation following non-pituitary procedures. Furthermore, signs and symptoms of hypopituitarism are often unspecific and may be masked by what has been assumed to be a “post-traumatic” or “post-operative syndrome”. The potentially life threatening condition of hypocortisolism, however, underscores the importance of screening for hypopituitarism after intracranial operations. Neurosurgeons and rehabilitation physicians should thus be aware of hypopituitarism and the screening modalities available.

It has been hypothesized that the anatomic location of the somatotrophic (in the lateral wing of the anterior lobe) and gonadotrophic cells (in the pars distalis and tuberalis), a vulnerable vascular region of the long hypophyseal portal system (passing through the sellar diaphragma), is responsible for the high rate of disturbances observed along these axes. In contrast, corticotrophic and thyrotrophic cells are located more anteromedially, in a more protected territory of the short hypophyseal portal system [2,4,9,27-29]. Apart from direct trauma, hypoperfusion of the pituitary gland may be the most common cause of postoperative disturbances [9,30,31]. Additionally, it has been postulated that a secondary hypothalamic disturbance following brain radiation in children is due to an altered neurotransmitter input from other brain centers [32].

In patients without a known history of seizures, we found a high rate (41.9%) of predominantly solitary adrenocorticotrophic deficiency using the ITT. In the eight patients with seizures, the low dose ACTH test was performed as an alternative. Of note, among these patients, we found that 87.5% had an adrenocorticotrophic deficiency. The explanation for this high rate remains unclear. False positive results must be kept in mind, although the low dose ACTH test used here has been noted to correlate highly with the ITT [33-35]. On the other hand, it might be possible that the rate of this hormonal insufficiency has been underestimated due to the absence of dynamic testing for the adrenocorticotrophic axis in other studies. Furthermore, seizure itself or accompanying seizure medication may result in hormonal disturbances. Overall, the detection of hormonal disturbances in patients with epileptic seizures differed significantly from detection in patients without a history of seizures (p = 0.0183).

### Limitations of the study

We did not perform preoperative endocrine evaluations. Furthermore, by testing at a single point in time, we were unable to differentiate definitively between permanent and intermittent dysfunction.

Furthermore, the timing of postoperative testing was variable and we did not perform testing on a control group. Due to the inclusion of many different types of pathologies in small groups it is unclear to know which disease processes are strongly associated with hypopituitarism.

## Conclusion

Despite the limitations described above, we found high prevalence of hypopituitarism in postoperative neurosurgery patients with non-pituitary operation procedures. Furthermore, a recent study indicates that hypopituitarism already exists before surgery as a frequent finding. Endocrine evaluation should be part of a pre- and postoperative screening protocol, at the very least in patients suffering from unexplained and diffuse complaints. We want to appeal to perform further prospective multicenter studies with a larger number of patients and with implementation of pre-operative testing. From our point of view guidelines for example with recommendations for optimal time points of postoperative testing are required.

## Abbreviations

ACTH: Adrenocorticotropic hormone; BMI: Body mass index; BW: Body weight; CI: Confidence interval; ETV: Endoscopic third ventriculostomy; fT4: Free thyroxin; FSH: Follicle-stimulating hormone; GH: Growth hormone; GHD: Growth hormone deficiency; GHRH: Growth hormone releasing hormone; IGF-I: Insulin-like growth factor-I; ITT: Insulin tolerance test (ITT-tolerance); LH: Luteinizing hormone; SAH: Subarachnoid hemorrhage; SD: Standard deviation; TBI: Traumatic brain injury; TSH: Thyroid-stimulating hormone; VP: Ventriculo-peritoneal.

## Competing interests

H. Wallaschofski received travel and research grants from Pfizer and Novo Nordisk. He is a member of the German as well as the International KIMS board. C. Spielhagen received travel grants from Pfizer and Novo Nordisk. S. Fleck received a travel grant from Pfizer and Novo Nordisk.

## Authors’ contributions

Each author contributed to the paper according to the ICMJE guidelines for authorship. SF contributed to study conception, collecting of data, interpretation of data, and drafting the manuscript. HW contributed to study conception, analysis and interpretation of data, and revising of the manuscript. CR has been involved in data acquisition and revision of the manuscript. MM contributed to analysis and statistical interpretation, and drafting the manuscript. TM has been involved in analysis and interpretation of data, and revision of manuscript. MN contributed to analysis and interpretation of data. HS has been involved in study conception, interpretation of data. CS has been involved in study conception, acquisitation and analysis of data, and drafting and revision of the manuscript. All authors have given final approval of the version to be published.

## Pre-publication history

The pre-publication history for this paper can be accessed here:

http://www.biomedcentral.com/1472-6823/13/51/prepub
